# Highly Sensitive and Selective Hydrogen Gas Sensor Using the Mesoporous SnO_2_ Modified Layers

**DOI:** 10.3390/s17102351

**Published:** 2017-10-14

**Authors:** Niuzi Xue, Qinyi Zhang, Shunping Zhang, Pan Zong, Feng Yang

**Affiliations:** 1 School of Material Science and Engineering, Wuhan University of Technology, Wuhan 430070, China; xueniu@whut.edu.cn (N.X.); Mrzongpan@163.com (P.Z.); yfeng047@163.com (F.Y.); 2 Department of Materials Science and Engineering, Huazhong University of Science and Technology, Wuhan 430074, China; pszhang@mail.hust.edu.cn

**Keywords:** gas sensor, mesoporous SnO_2_, hydrogen, sensitivity, selectivity

## Abstract

It is important to improve the sensitivities and selectivities of metal oxide semiconductor (MOS) gas sensors when they are used to monitor the state of hydrogen in aerospace industry and electronic field. In this paper, the ordered mesoporous SnO_2_ (m-SnO_2_) powders were prepared by sol-gel method, and the morphology and structure were characterized by X-ray diffraction analysis (XRD), transmission electron microscope (TEM) and Brunauer–Emmett–Teller (BET). The gas sensors were fabricated using m-SnO_2_ as the modified layers on the surface of commercial SnO_2_ (c-SnO_2_) by screen printing technology, and tested for gas sensing towards ethanol, benzene and hydrogen with operating temperatures ranging from 200 °C to 400 °C. Higher sensitivity was achieved by using the modified m-SnO_2_ layers on the c-SnO_2_ gas sensor, and it was found that the S(c/m2) sensor exhibited the highest response (Ra/Rg = 22.2) to 1000 ppm hydrogen at 400 °C. In this paper, the mechanism of the sensitivity and selectivity improvement of the gas sensors is also discussed.

## 1. Introduction

As one of the most important clean energies, H_2_ is widely used in various fields such as fuel cell vehicles, aerospace industry, petrochemical industry, and electronic field [1,2,3]. In consideration of the leakage in the applications of H_2_ whose explosive limit is very low, it is essential to monitor the state of hydrogen. Gas sensor is one of the most effective detectors [4,5]. 

Great emphasis is being given to metal oxide semiconductors (MOS), including ZnO [6], WO_3_ [7], TiO_2_ [8], In_2_O_3_ [9] and SnO_2_ [10], as gas sensing materials for a long time. Among various MOS gas sensors, SnO_2_-based gas sensors are widely used because of their low cost, high sensitivity and long-term stability [11]. However, poor selectivity to various gases restricts their applications. Gas sensing performances of SnO_2_, especially the selectivity to H_2_, can be improved by applying doping [12,13,14], catalyst [15,16,17], filtering membranes [18,19,20], etc. For example, Inyawilert et al. studied the films of SnO_2_ nanoparticles doped with 0.1~2 wt.% rhodium (Rh). It showed that the Rh-doped SnO_2_ sensor presented high H_2_ selectivity against NO_2_, SO_2_, C_2_H_4_, C_3_H_6_O, CH_4_, H_2_S and CO [13]. Liewhiran et al. reported that Pd-catalyzed SnO_2_ sensor (0.2 wt.% Pd/ SnO_2_, 10 μm in thickness) showed ultra-high response to H_2_ [15]. It was found in the work of Tournier et al. that SiO_2_ filter film deposited on the SnO_2_ film is highly selective to hydrogen [18]. Filtering membranes such as SiO_2_, Al_2_O_3_, Fe_2_O_3_, etc. work as molecular sieves. They are useful to improve the selectivity of gas sensors.

Gas sensing performances of SnO_2_ gas sensors can be highly improved by using mesoporous material because of its high specific surface area (SSA) [21]. Japanese researchers fabricated nano-SnO_2_ powders coated by mesoporous SnO_2_ (m-SnO_2_), and this kind of SnO_2_ films highly increased the responses to H_2_ [21]. It was found in the work of Pijolat et al. that thin SiO_2_ films deposited on the SnO_2_ thick films could improve the selectivity to H_2_ [22]. Dhawale et al. synthesized mesoporous ZnO thin films which showed high selectivity towards liquefied petroleum [23]. The aim of the present study was to improve the selectivity and sensitivity simultaneously in hydrogen detection. The uses of such mesoporous materials enable enhancement of the adsorption and reaction of test gas because of the high specific surface area. On the other hand, mesoporous materials are potential molecular sieves for gas sensors to improve their selectivity because of the mesoporous structure.

In this paper, the m-SnO_2_ powders were synthesized with a simple and low cost sol-gel method. The sensors were fabricated using commercial SnO_2_ (c-SnO_2_) films as the basic layer and the m-SnO_2_ films as the modified layers by screen printing method. Their sensing performances were tested with hydrogen, ethanol and benzene. The relationships between selectivity and the thickness of the films were studied. The present study aims to develop a low cost and highly sensitive and selective hydrogen gas sensor.

## 2. Materials and Methods

### 2.1. Preparation of m-SnO_2_ Powders

Employing Na_2_SnO_3_·4H_2_O as the Sn source, n-cetylpyridinium chloride (C_16_PyCl) as the template and trimethylbenzene (C_6_H_3_(CH_3_)_3_) as the surfactant, m-SnO_2_ powders were prepared in a similar way to that reported previously [21]. The typical preparation manner was as follows. C_16_PyCl was added to the deionized water at 2.6 wt.%, while Na_2_SnO_3_·4H_2_O was dissolved in the deionized water at 3.6 wt.%. In this case, Na_2_SnO_3_·4H_2_O aqueous was mixed with the C_16_PyCl solution at a molar ratio [C_16_PyCl]/[Na_2_SnO_3_·4H_2_O] = 2.0. Then, trimethylbenzene was added to the solution at a molar ratio [C_6_H_3_(CH_3_)_3_]/[Na_2_SnO_3_·4H_2_O] = 2.5. The pH of the mixture was then adjusted to 10 with an aqueous 35 wt.% HCl solution. The resultant emulsion solution was aged for 2 days at 25 °C. After suction filtration with deionized water and drying, the resultant solid products were treated with a 0.1 M aqueous phosphoric acid (PA) solution for 2 h with magnetic stirrers. Then, it was filtered off, washed and dried at 60 °C for 12 h. Eventually, the solid was calcined at 600 °C for 5 h in air. After calcination, the powders were subjected to mechanical grinding with an agate mortar.

The crystal phases of the m-SnO_2_ powders were characterized via X-ray diffraction analysis (XRD, D8 Adwance, Bruker, Karlsruhe, Germany). The specific surface area and pore size distribution were measured by the Brunauer–Emmett–Teller (BET) method using a N_2_ adsorption isotherm (BET, ASAP 2020, Micromeritics, Norcross, GA, USA). Morphology of the m-SnO_2_ powders was observed by a transmission electron microscope (TEM, JEM2100F STEM/EDS, JEOL, Tokyo, Japan) and the morphology of the commercial SnO_2_ (c-SnO_2_) powders was observed by a scanning electron microscope (SEM, JSM-IT300, JEOL, Tokyo, Japan).

### 2.2. Fabrication of SnO_2_ Sensors

**Figure 1:**
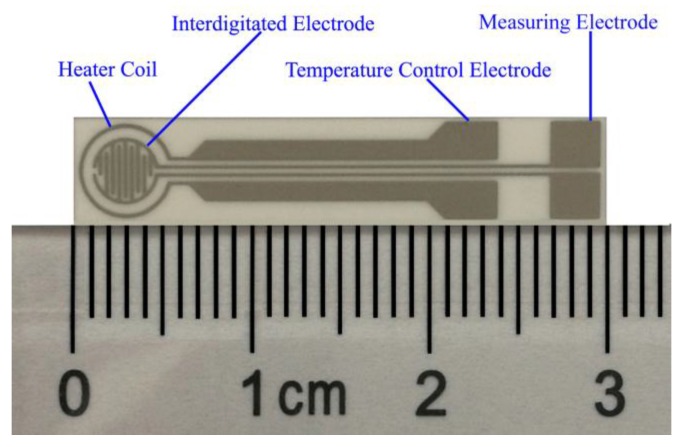
The substrate structure of the gas sensor.

Pastes of the c-SnO_2_ powders and the as-prepared m-SnO_2_ powders were applied on a substrate (30 mm × 6 mm × 0.625 mm), on which interdigitated Pt electrodes had been printed with mechanically automated screen printing technology, as shown in Figure 1.

The thick film gas sensors were fabricated using screen printing technology. For the first layer, the c-SnO_2_ powders were mixed with the printing oil (YY-1010, Wuhan Huachuang Ruike Tech. Co. LTD, Wuhan, China) at the mass ratio of 1:1 as the paste. Furthermore, to improve the stability of the gas sensors, the frit of PbO, B_2_O_3_, and SiO_2_ (mass ratio [PbO]/[B_2_O_3_]/[SiO_2_] = 45/35/20) was added into the c-SnO_2_ powders at the level of 2 wt.%. The substrates were treated with drying at room temperature for 10 min and 50 °C for 1 h when the pastes were printed on them. For modified layer, the paste was mixed with the m-SnO_2_ powders and the printing oil at the same mass ratio of 1:1. To prepare more modified layers, simply repeat the printing step above. Eventually, the gas sensors were dried at 50 °C for 1 h and calcined at 650 °C for 2 h. The different fabricated gas sensors are listed in Table 1.

The surface morphology of the prepared gas sensors was observed by a scanning electron microscope (SEM, Zeiss Utral Plus, Cari Zeiss AG, Jena, Germany). The cross-sections of the different SnO_2_ films were observed by a scanning electron microscope (SEM, S-4800, HITACHI, Tokyo, Japan).

### 2.3. Measurement of Sensing Performance

**Figure 2:**
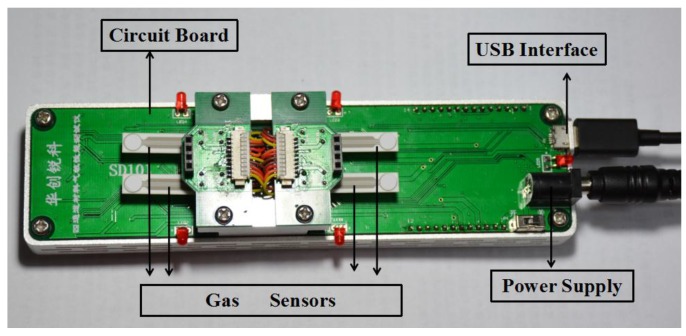
The SD-101 gas sensing performance testing device.

The gas sensors were measured by a commercial SD-101 gas sensing performance testing device (Wuhan Huachuang Ruike Tech. Co. LTD, Wuhan, China) which can be used with four gas sensors to test their gas sensing performance simultaneously (Figure 2). The operating temperature can be controlled via adjusting the power of the heater coil by a microprocessor. The operating temperature of the gas sensors is in the range of room temperature to 450 °C.

**Figure 3:**
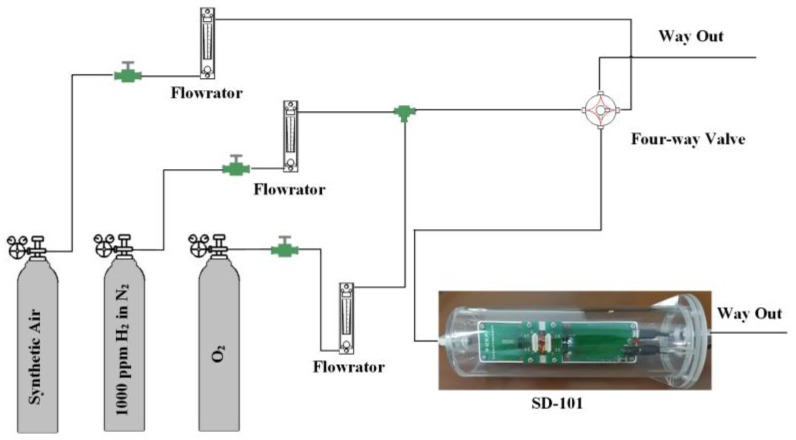
Gas sensing testing device.

The prepared gas sensors were measured to sense 1000 ppm H_2_ with dynamic method and 10 ppm ethanol and benzene with static method at the temperature of 200 °C, 250 °C, 300 °C, 350 °C and 400 °C. In the process of dynamic measurement, the SD-101 gas sensing performance testing device was placed in a cylinder of 60 mm in diameter, which is made of polymethyl methacrylate (PMMA). The testing gas flowchart is shown in Figure 3.

**Figure 4:**
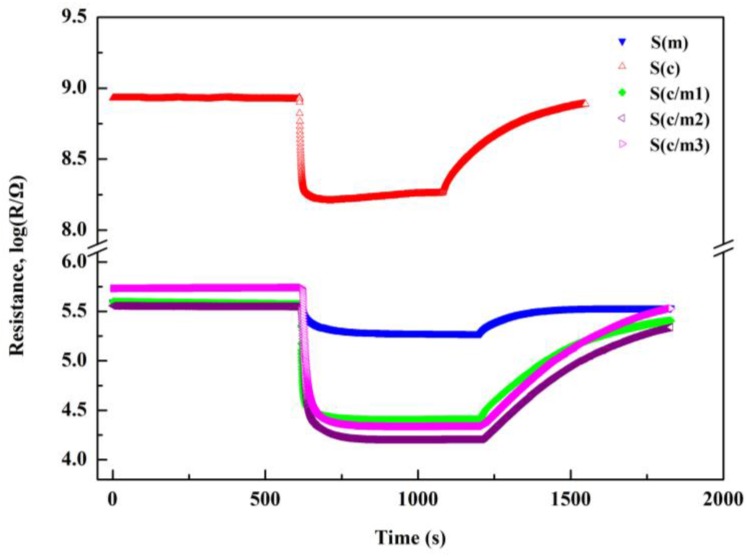
Response transients of the gas sensors to 1000 ppm H_2_ at 400 °C.

The synthetic air, whose flow rate was set as 250 mL/min, consisted of N_2_ and O_2_ at the volume ratio of 4:1. To match with the synthetic air, the volume ratio of the 1000 ppm H_2_ in N_2_ and O_2_ was also set as 4:1 with the flow rate of 200 mL/min and 50 mL/min, respectively. During the testing process, the synthetic air was replenished by adjusting the four-way valve. The four-way valve is first turned to let the hydrogen in when the response was stabilized. When the response was stabilized, the four-way valve is turned to lead the synthetic air to go through the cylinder until the sensors recover from the hydrogen. The response transients of the gas sensors to 1000 ppm H_2_ at 400 °C is shown in Figure 4. It is obvious that all the gas sensors exhibit stable and quick response. In the process of dynamic measurement, the SD-101 gas sensing performance testing device was placed in a cubic evaporated cavity for 50 L. During the testing process, the corresponding quantities of the organic solution (ethanol and benzene) were injected by a micro-injector on a heating panel in the evaporated cavity, when the gas sensors responses to air stabilized. When the response to the test gas stabilized, the cubic testing cavity was opened for recovery.

The response is defined as Ra/Rg, where Ra and Rg are the sensor resistances in air and in the test gas, respectively. The response time is generally defined as the time necessary for achieving a 90% resistance change to the steady-state value. The recovery time is defined as the time for sensor resistance to reach 90% of air resistance.

## 3. Results and Discussion

### 3.1. Characterization of the c-SnO_2_ Powders and the m-SnO_2_ Powders

**Figure 5:**
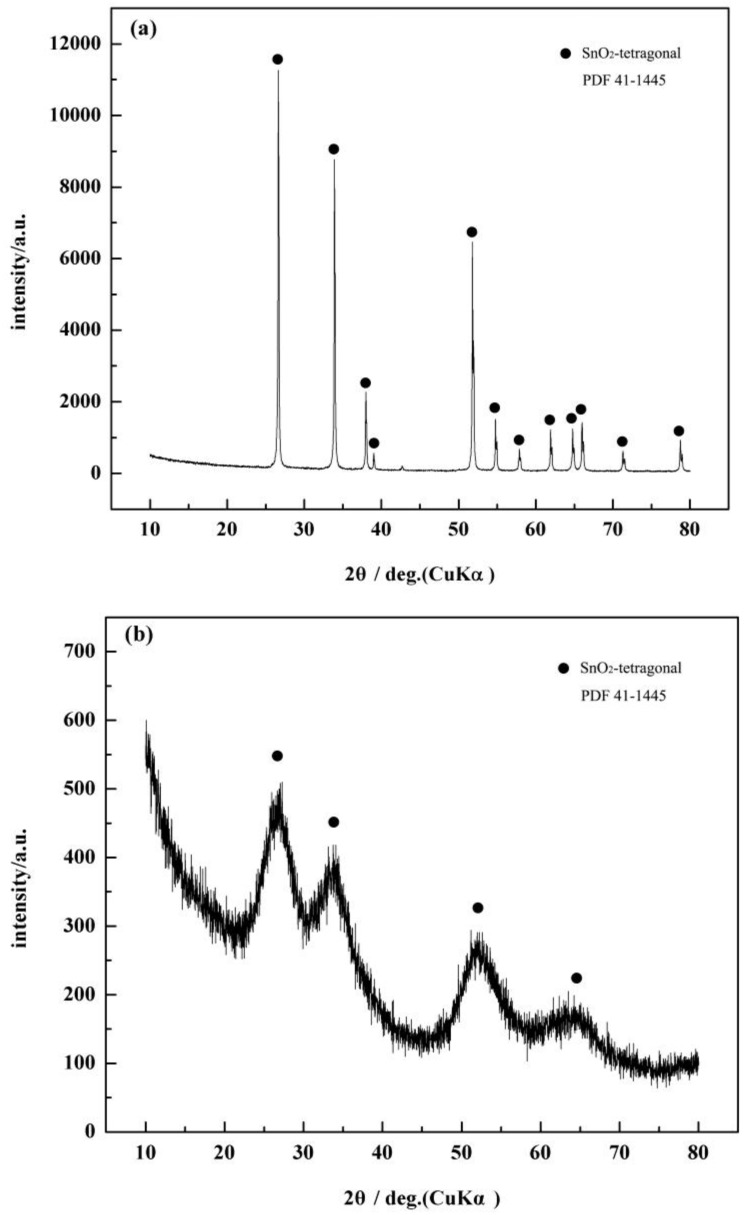
XRD patterns of the c-SnO_2_ powders and the m-SnO_2_ powders.: (**a**) c-SnO_2_; and (**b**) m-SnO_2_.

**Figure 6:**
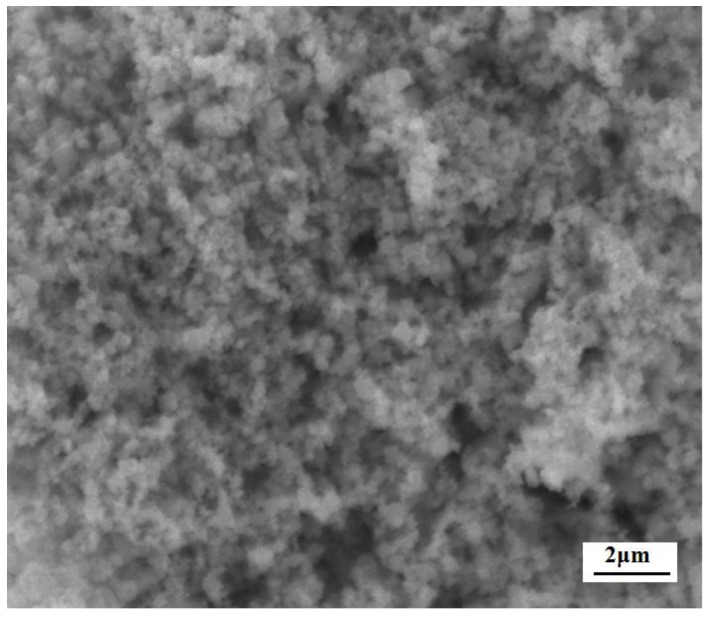
SEM image of the c-SnO_2_ powders.

**Figure 7:**
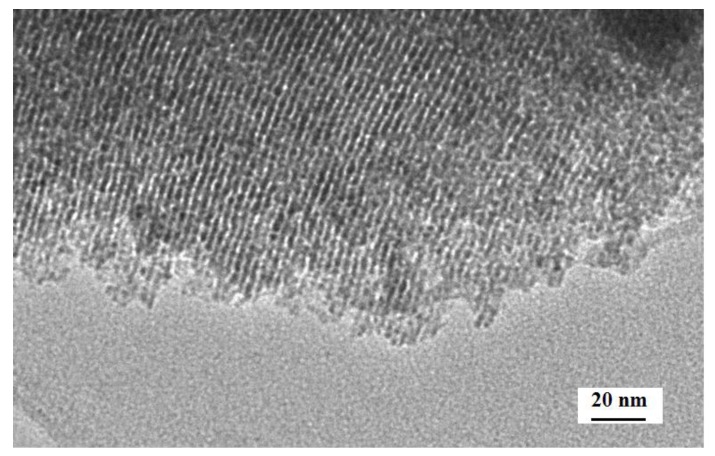
TEM image of the m-SnO_2_ powders.

**Figure 8:**
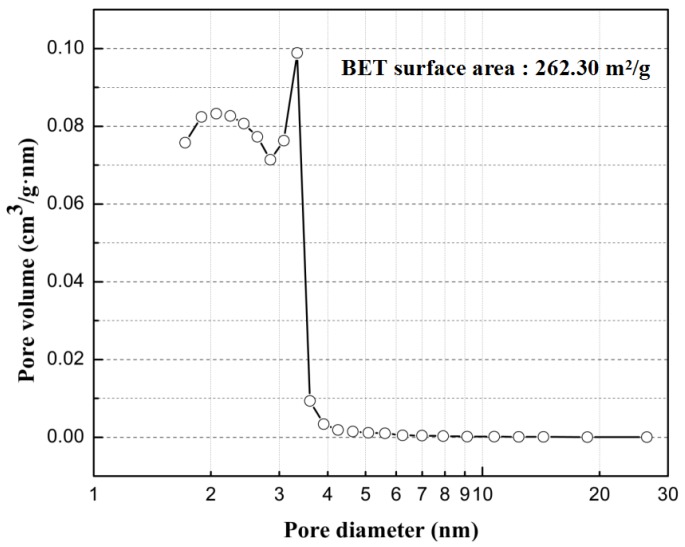
Pore size distribution of the m-SnO_2_ powders.

Figure 5 shows XRD patterns of the c-SnO_2_ powders (Figure 5a) and the m-SnO_2_ powders (Figure 5b). The c-SnO_2_ powders have peaks corresponding to the SnO_2_ crystalline phase (PDF 41-1445). This implies that the c-SnO_2_ powders are well-crystallized, and have a tetragonal SnO_2_ phase. The crystallite size of the c-SnO_2_, calculated by Scherrer’s equation (Jade), is about 65.5 nm. It is also confirmed by the SEM image (Figure 6). The XRD pattern of the m-SnO_2_ powders (Figure 5b) shows that they have some main peaks corresponding to SnO_2_ crystalline phase. It reveals that the prepared m-SnO_2_ powders have low crystallinity. In addition, the ordered mesoporous structure is confirmed clearly by the TEM observation of the m-SnO_2_ powders in Figure 7. The pore size distribution and the specific surface area of the m-SnO_2_ powders are shown in Figure 8. It is clear that the m-SnO_2_ powders show a large SSA of 262.30 m^2^/g with a small pore size of 2.6 nm.

### 3.2. Characterization of Gas Sensors

**Figure 9:**
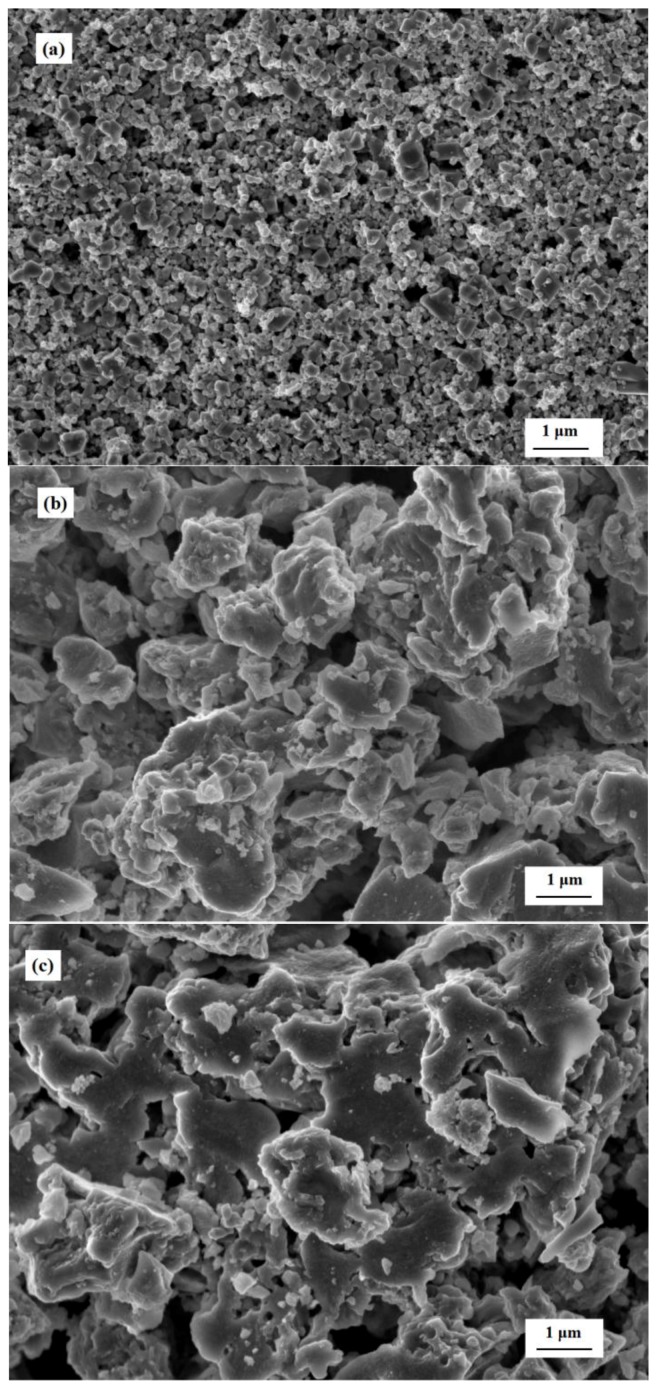

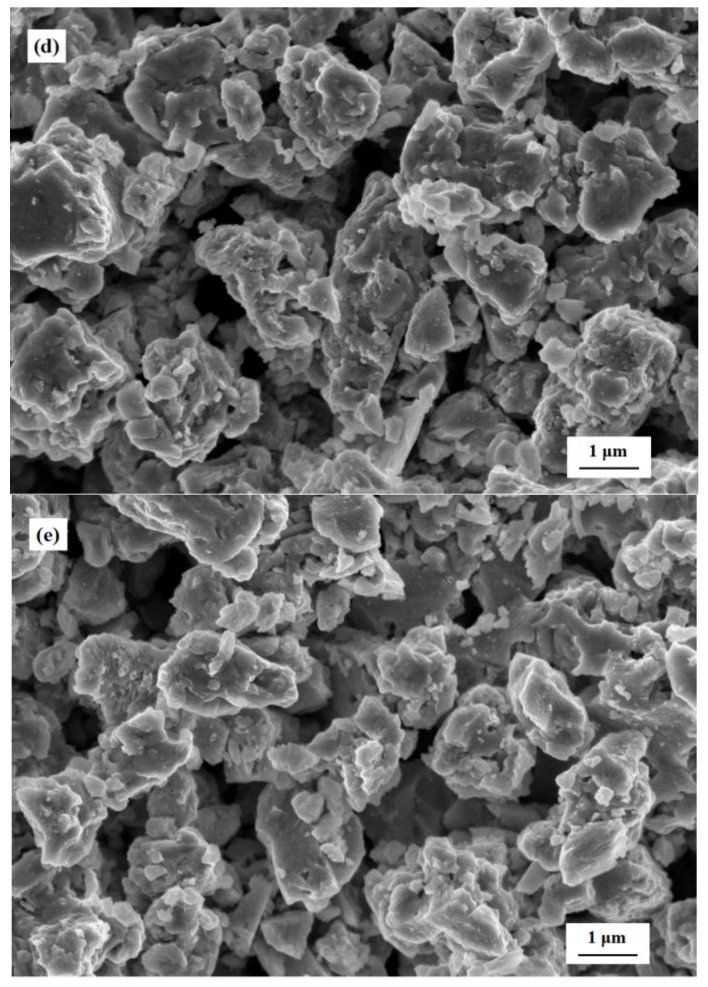
SEM images of the surfaces of the gas sensors: (**a**) S(c); (**b**) S(m); (**c**) S(c/m1); (**d**) S(c/m2); and (**e**) S(c/m3).

Figure 9 shows the SEM images of the surface morphology of the gas sensors. It was found that the basic layers of the S(c) sensor were dense and have flat surfaces (see Figure 9a). Moreover, the S(c) sensor appeared to have a particle size of approximately dozens of nanometers, despite a small quantity of lager particles. The calcination resulted in some sintered macropores with a size of several hundred nanometers. In contrast, the film of the S(m) sensor show rough and loosened surfaces, as shown in Figure 9b. It is obvious that the S(m) sensor film showed lager particles (100–200 nm) than that of the S(c) sensor film due to the agglomerations of the particles. The agglomerations of the m-SnO_2_ were extremely distinct from those of c-SnO_2_. Furthermore, the calcination of the S(m) sensor resulted in lager sintered macropores. The surface morphology of the other sensors is similar to those of the S(m) sensor (see Figure 9c–e) because of the same printing materials and printing process.

**Figure 10:**
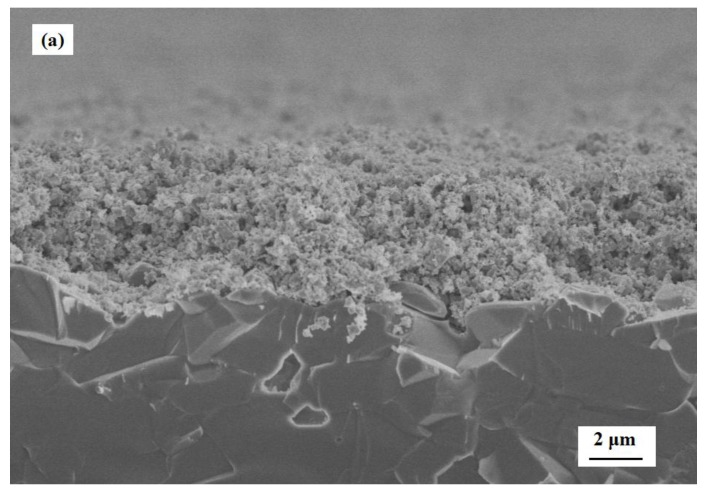

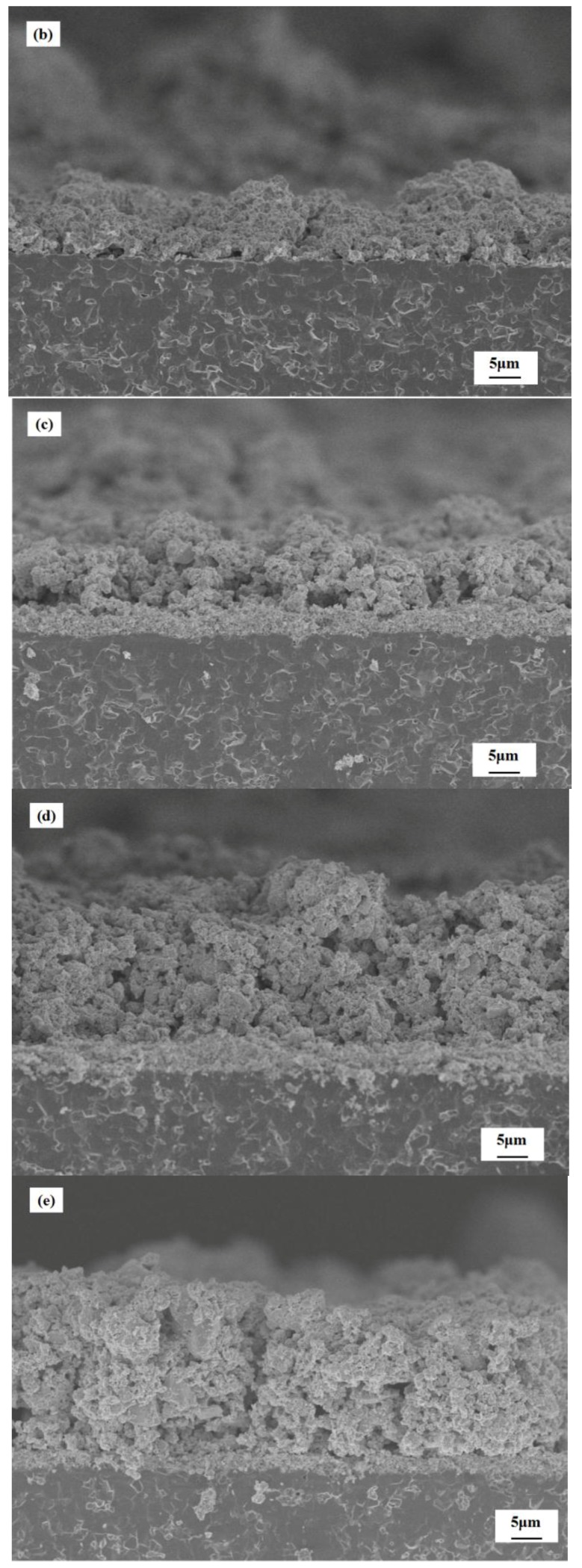
SEM images of the cross-section of the gas sensors: (**a**) S(c); (**b**) S(m); (**c**) S(c/m1); (**d**) S(c/m2); and (**e**) S(c/m3).

The SEM images of the cross-sections of the gas sensors are shown in Figure 10. The cross-sectional morphology of the S(c) sensor shows that the calcined c-SnO_2_ was more compact with the thickness of about 5 μm (see Figure 10a), but the modified layers of the m-SnO_2_ showed relatively loosened morphology (see Figure 10b). It is apparent that there is an obvious stratification between the c-SnO_2_ basic layer and the m-SnO_2_ modified layer (see Figure 10c). In addition, fabricated with the same materials and printing manner, the m-SnO_2_ modified layers had no stratification to each other. The thickness of each m-SnO_2_ modified layer was confirmed with SEM observation to be about 10–15 µm. Thus, the thickness of the m-SnO_2_ modified layers of the S(c/m1), S(c/m2) and S(c/m3) sensors were confirmed to be 15 µm, 31 µm and 41 µm, respectively (see Figure 10c–e).

### 3.3. The Resistance of the Gas Sensors in Air

**Figure 11:**
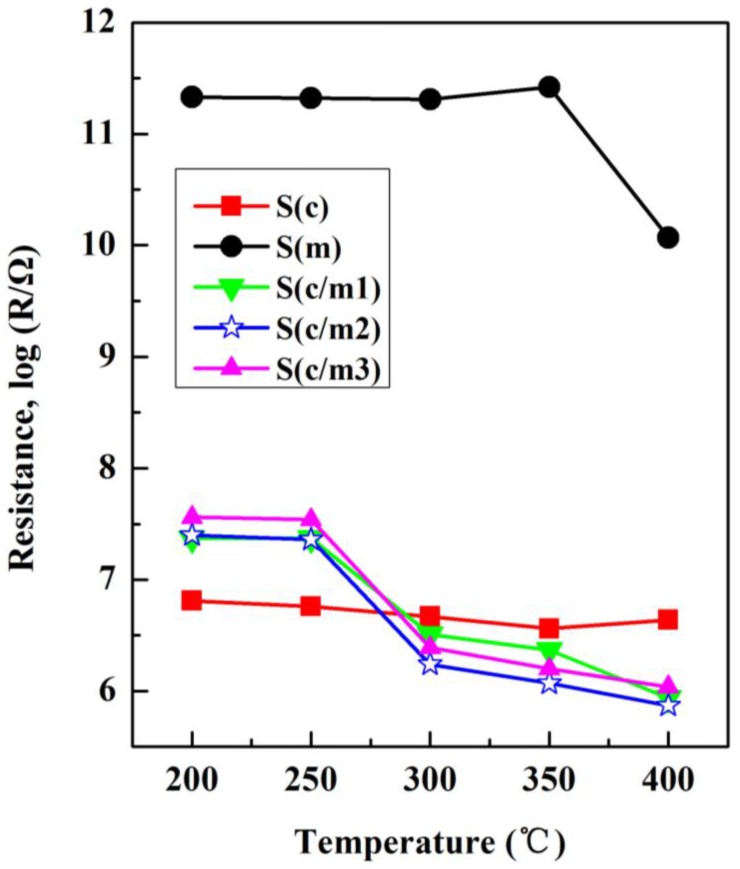
Temperature dependence of the resistances of the gas sensors in air.

Figure 11 shows the temperature dependence of the resistances of the gas sensors in air. As a semiconductor material, the resistance of SnO_2_ shows decrement trend due to the increase of carriers at the condition of thermal excitation, as confirmed in Figure 11. The higher is the operating temperature in which the gas sensors work, the lower is the resistances in air.

The values of the resistance in air of the S(c) sensor were slightly decreased due to the frit. The resistance of the S(m) sensor in air was much higher, which leads to difficult measurement problem in its application. It can be ascribed to the mesoporous structure, which leads to the extreme decrease of conductive path [21]. However, the resistance of the S(m) sensor in air decreased obviously when the operating temperature increased to 400 °C, owing to the condition of thermal excitation [24]. Using the m-SnO_2_ as the modified layers, the resistances of the (S(c/m1), S(c/m2) and S(c/m3)) sensors changed obviously. Since the resistance of SnO_2_ semiconductors was affected by thermal excitation, the resistance of the (S(c/m1), S(c/m2) and S(c/m3)) sensors in air decreased remarkably when the operating temperature increased to 300 °C. As for the S(c/m1), S(c/m2) and S(c/m3) sensors, the resistance of the S(c/m2) sensor in air appeared to be the lowest at all the tested operating temperature. The value of the resistance in air of the S(c/m2) sensor was 3.7 × 10^5^ Ω at 400 °C. The above results demonstrate that both thermal excitation and adsorption affect the resistance of MOS gas sensors in air. The oxygen adsorbates were considered as the main reason to change the resistance of MOS gas sensors in air. The absorbed O_2_ on the surface of SnO_2_ films implies the formation of O^−^ or O^2−^, which result in a decrease in the quantity of carrier. Thus, the resistance of the sensors fabricated with the modified m-SnO_2_ layers in air increased, due to the marked improvement of the adsorption capacity of surface oxygen. However, since the increase of the modified m-SnO_2_ layers resulted in larger distance for the oxygen adsorbates diffusing to the basic c-SnO_2_ layer, as well as the diffusion inhibition of mesoporous to oxygen, the S(c/m3) sensor showed lower resistance in air than the S(c/m2) sensor.

### 3.4. Sensing Responses to the Testing Gas

**Figure 12:**
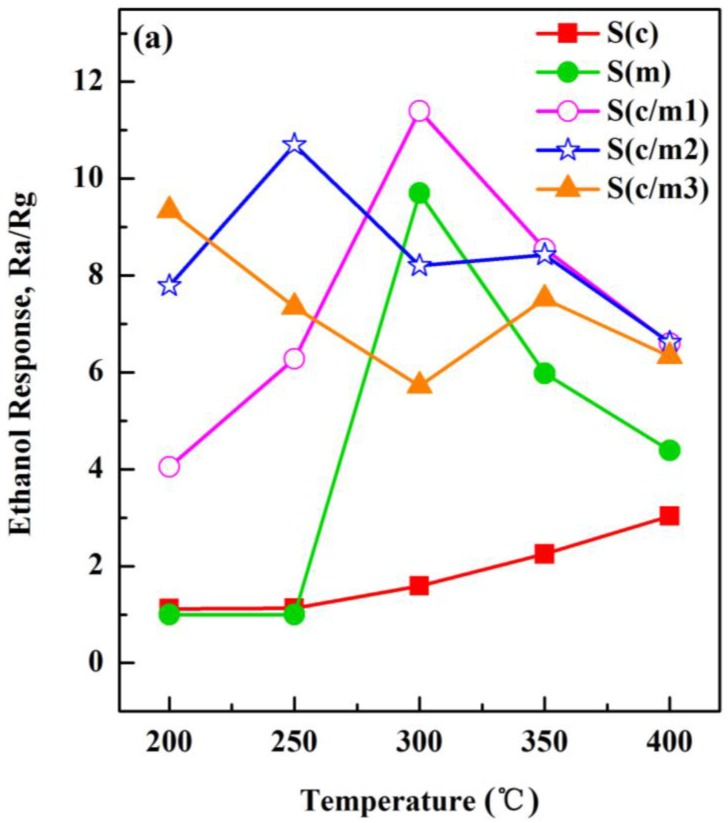

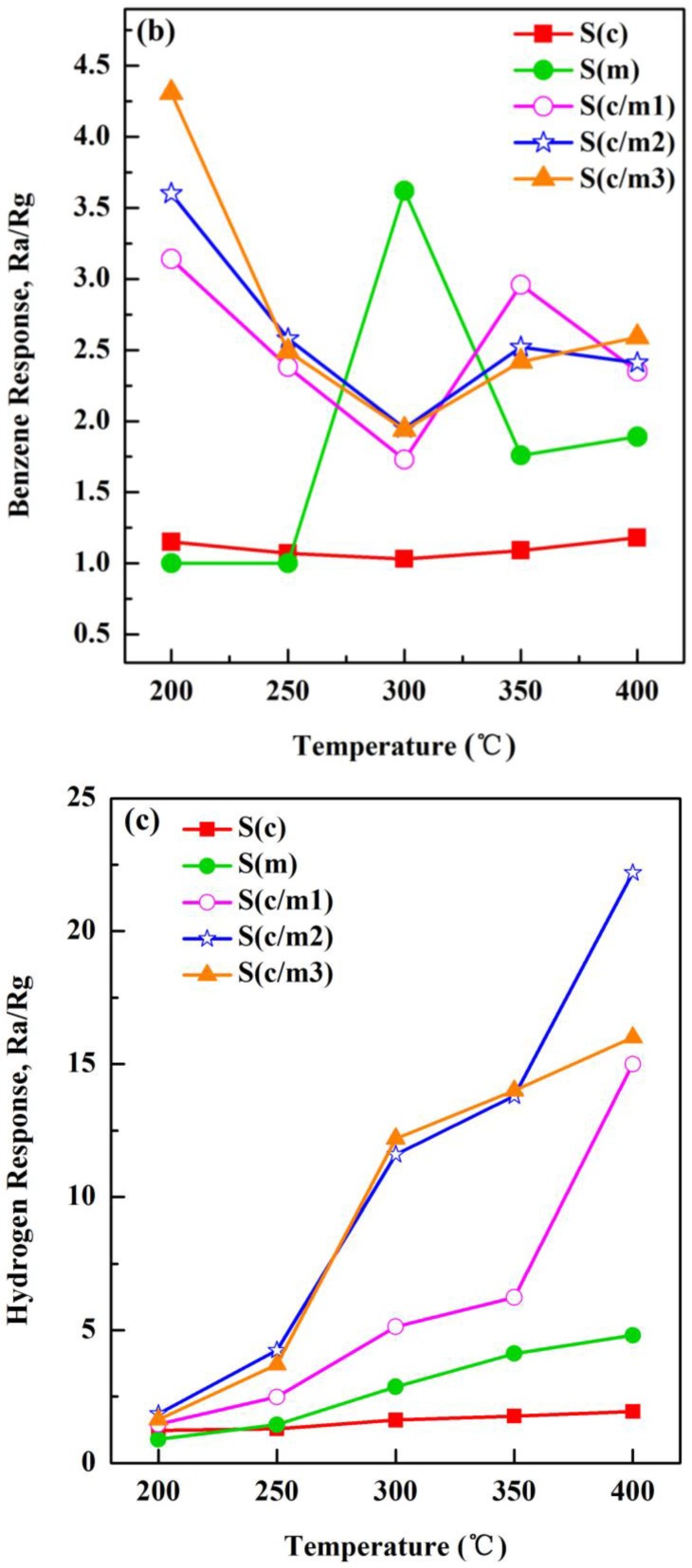
Temperature dependence of the responses of the gas sensors: (**a**) ethanol at 10 ppm; (**b**) benzene at 10 ppm; and (**c**) hydrogen at 1000 ppm.

The temperature dependence of the responses to ethanol, benzene and hydrogen are depicted in Figure 12. The response of the S(c) sensor to ethanol at 10 ppm increased slightly with the increasing of operating temperature up to 400 °C, as shown in Figure 12a. However, the responses to ethanol of the S(c) sensor were lower than those of the S(c/m1), S(c/m2) and S(c/m3) sensors, due to the large specific surface area (262.30 m^2^/g) of the m-SnO_2_. In addition, the S(c/m3) sensor showed the largest response (Ra/Rg = 11.4) to ethanol at 300 °C. The responses to benzene of the S(c/m1), S(c/m2) and S(c/m3) sensors showed a similar tendency: the response decreased at the relatively low operating temperature due to a slight effect of thermal diffusion. While the reaction between testing gas and the basic c-SnO_2_ was controlled by gas absorption, the response to benzene was improved with the increasing of operating temperature from 300 °C to 400 °C mainly due to the thermal diffusion. The S(c/m3) sensor exhibited the largest response (Ra/Rg = 4.31) to benzene at 200 °C, as shown in Figure 12b. The response to hydrogen increased from 200 °C to 400 °C (Figure 12c), while the S(c/m2) sensor showed the largest response (Ra/Rg = 22.2) to hydrogen at 400 °C. It can be deduced from the hydrogen molecular diffusion that the small molecular dimension of hydrogen benefits the gas diffusion. While gas adsorbing capacity was enhanced, the response to hydrogen was highly improved (see Figure 12c). All of the above results confirmed that the modified layers of the m-SnO_2_ contribute to improving the response of the S(c) sensor sufficiently. Moreover, the magnitude of the response enhancement is not directly proportional to the amount of the m-SnO_2_.

**Figure 13:**
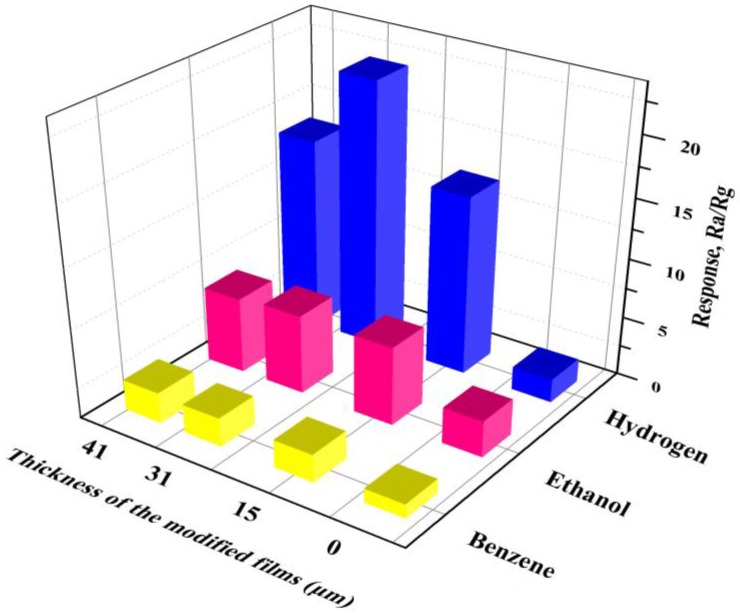
Evolutions of the response versus thickness of the modified films of the gas sensors at 400 °C. The thickness of the modified films with the gas sensors: S(c), 0 μm; S(c/m1), 15 μm; S(c/m2), 31 μm; and S(c/m3), 41 μm.

To further investigate the effects of the modified m-SnO_2_ layers, the evolutions of the response versus thickness of the modified films of the gas sensors at 400 °C are depicted in Figure 13. It is clear that the response of the S(c/m1), S(c/m2) and S(c/m3) sensors to ethanol, benzene and hydrogen were all improved to a certain extent in comparison to the S(c) sensor response, which means higher gas sensitivities. Especially, the response of the S(c/m2) sensor to hydrogen appeared to improve by 11.4 times, compared to benzene and ethanol improvements of 2.03 and 2.18 times, respectively.

### 3.5. The Response and Recovery Times of the Gas Sensors

Table 2 shows the response and recovery times of the gas sensors to ethanol, benzene and hydrogen. The response and recovery times of some of the gas sensors were difficult to summarize due to the lower response at low operating temperature. The response time of the S(c) sensor to ethanol was markedly short from 350 °C (response time = 115 s) to 400 °C (response time = 54 s), while the values of response time to hydrogen at 350 °C and 400 °C were 80 s and 89 s, respectively. However, the response times of benzene were hard to summarize because of the relatively large molecular dimension (0.65–0.68 nm), which led to the low response to benzene. In the case of the S(c/m1), S(c/m2) and S(c/m3) sensors, the response times increased with the thicker modified m-SnO_2_ layers for all of the tested gases at 350 °C and 400 °C. In addition, the smallest response time appeared to be 74 s with the S(c/m1) sensors to hydrogen at 400 °C. It can be ascribed to the large specific surface area and the molecular diffusion at high temperature, both of which lead to easy gas diffusion inside the mesopores.

All gas sensors showed longer recovery times to ethanol and benzene in comparison to hydrogen from 350 °C to 400 °C. Especially, the S(c/m1), S(c/m2) and S(c/m3) sensors tended to show a longer response time (>500 s) to hydrogen than to ethanol (<467 s) and benzene (<207 s).

### 3.6. Discussion

**Figure 14:**
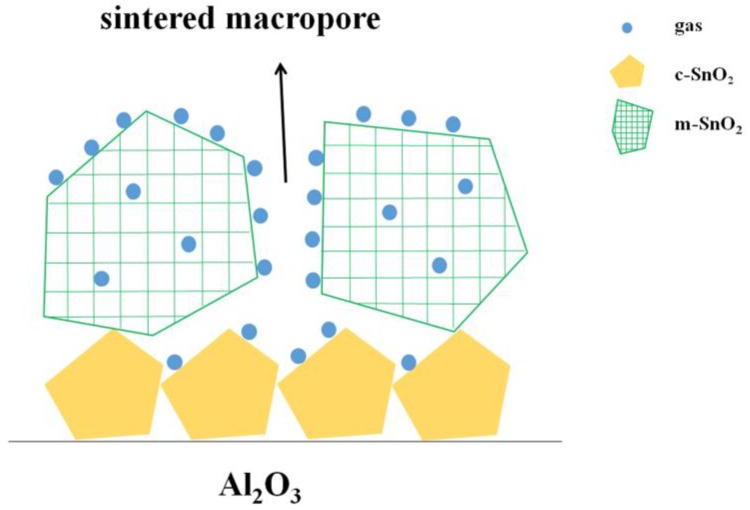
Schematic drawing of the possible gas sensing mechanism of the gas sensors.

Among these, the possible gas sensing mechanism of the gas sensors are shown in Figure 14. It is considered that the adsorption/desorption properties of the mesoporous influenced the gas sensing performances of the gas sensors. Owing to the large specific surface area of m-SnO_2_, which could enhance the adsorption of gas molecules, the S(c/m1) S(c/m2) and S(c/m3) sensors exhibited higher gas (ethanol, benzene, and hydrogen) responses than those of the S(c) sensor. The responses of benzene are lower than those of ethanol and hydrogen because of its weak reducibility and larger size of benzene ring, which is difficult to pass through the m-SnO_2_ modified layer. However, the sintered macropores (Figure 14) among the m-SnO_2_ is helpful to adsorb more benzene molecules. This is why the responses of the S(c/m1), S(c/m2) and S(c/m3) sensors to benzene are higher than those of the S(c) sensor to benzene. In contrast, the smaller molecular size of the hydrogen is beneficial to pass through the ordered structure of m-SnO_2_ and the sintered macropores. Thus, thickness of the films and the ordered level of mesoporous influenced the gas sensing performance of the gas sensors fabricated with the modified m-SnO_2_ layers. In addition, further approaches to control the amount of sintered macropores, the thickness of the films, surface contact of the films and the ordered level of mesoporous would be effective to improve the sensing performance of the gas sensors.

Experimental results of SnO_2_ sensors have been compared with the results reported by the other researchers on H_2_ sensors. Manjula et al. reported the Pd doped m-SnO_2_ gas sensors. It showed that the 0.25% Pd doped gas sensor response towards 1000 ppm hydrogen at 50 °C is 0.95. The gas sensors showed zero response to ethanol, LPG, NH_3_ and acetone [25]. Seftel et al. obtained gas sensing material by combining Pt with SnO_2_ or In_2_O_3_ based on SBA-15. The response of the gas sensor based on Pt/SnO_2_/SBA-15 is about 1.4 to 1000 ppm hydrogen at 350 °C [26]. Although the selectivity of the sensors was improved by doping, the responses of the sensors to hydrogen are no more than 2, which limits the applications of the sensors in hydrogen measurement.

We can find a large number of examples of sensitivities improvement of gas sensors to hydrogen when mesoporous structures are employed. Shen et al. reported that the influence of the different morphology of SnO_2_ nanomaterials on hydrogen sensing properties. They obtained the response of about 2.1 to 1000 ppm hydrogen at 250 °C for nanofilms [27]. Yeow et al. reported the gas sensors based on SnO_2_ nanospheres with various degrees porosity. The reference (SSA _SnO_2__ = 101.4 m^2^/g) gas sensor showed the largest response: 5.2 to 500 ppm hydrogen at 350 °C [28]. Zhao et al. prepared ordered mesoporous SnO_2_ and mesoporous Pd/SnO_2_ via nanocasting method using the hexagonal mesoporous SBA-15 as template. The maximum response of the sensor based on the ordered mesoporous SnO_2_ is 16.4 to 1000 ppm hydrogen at 300 °C [29]. Hayashi et al. prepared SnO_2_ gas sensors based on various m-SnO_2_ powders from two kinds of combination of tin source and surfactant template. The largest response of the gas sensors to 1000 ppm hydrogen at 350 °C appeared to be 42 [30]. It is evidential that the responses of the sensors based on the ordered mesoporous SnO_2_ to hydrogen have been dramatically increased. The one limitation of these studies is that the selectivity of the mesoporous SnO_2_ has not been studied.

Shahabuddin et al. reported the sputter deposited SnO_2_ thin film gas sensors with 9 nm thin Pt clusters. The Pt/SnO_2_ sensor shows an improvement in sensing response: 168 towards 500 ppm of hydrogen at 110 °C. The sensor revealed negligible cross sensing signals against acetone, IPA, NO_2_, methane, LPG, etc. [10]. Gong et al. reported the mesoporous nanocrystalline SnO_2_ gas sensor based on the fabricated SnO_2_ sputtering with Pt thin film. The gas sensor showed the response of about 1.8 to 1000 ppm hydrogen at 250 °C [31]. The sensitivities and selectivity of the SnO_2_ gas sensors could be significantly improved by sputtering with Pt thin film. However, the method requires an expensive facility and complex sample preparing process.

In our work, the ordered mesoporous SnO_2_ was prepared by simple sol-gel method. The gas sensors were prepared with a simple and low cost screen printing method while the mesoporous SnO_2_ worked as the modified layers. It was shown that both the sensitivities and the selectivity of the gas sensors to hydrogen were improved. The S(c/m2) sensor showed the largest response 22.2 to 1000 ppm hydrogen at 400 °C. The response to hydrogen is >10 times higher than that of the sensor without the modified layer (the S(c) sensor). Compared with the responses of the S(c) sensor, the responses of the S(c/m2) sensor to benzene and ethanol did not change significantly.

## 4. Conclusions

Ordered mesoporous SnO_2_ powders were prepared by employing Na_2_SnO_3_·4H_2_O, C_16_PyCl and trimethylbenzene. The specific surface area of the m-SnO_2_ powder was 262.30 m^2^/g after calcination at 600 °C. The gas sensors were fabricated using m-SnO_2_ films as the modified layers. It was proven that the gassensing performance of the gas sensors could be highly improved, especially to hydrogen, compared with ethanol or benzene gas. In addition, the S(c/m2) sensor exhibited the highest sensitivity (response: Ra/Rg = 22.2) to 1000 ppm hydrogen at 400 °C. The main reason for the high selectivity may be the diffusivity of hydrogen molecules in the ordered mesopores is easier than that of ethanol and benzene molecules.

## Figures and Tables

**Table 1:** The structure of the gas sensors.

Sample	Basic Layer	Modified Layer	Description
	Material	Material	
S(c)	c-SnO_2_	/	one layer of c-SnO_2_
S(m)	m-SnO_2_	/	one layer of m-SnO_2_
S(c/m1)	c-SnO_2_	m-SnO_2_	one layer of c-SnO_2_ and one layer of m-SnO_2_
S(c/m2)	c-SnO_2_	m-SnO_2_	one layer of c-SnO_2_ and two layers of m-SnO_2_
S(c/m3)	c-SnO_2_	m-SnO_2_	one layer of c-SnO_2_ and three layers of m-SnO_2_

**Table 2:** The response and recovery times of the gas sensors to ethanol, benzene and hydrogen.

Gas	Temperature (°C)	Response Time (s)					Recovery Time (s)				
		S(c)	S(m)	S(c/m1)	S(c/m2)	S(c/m3)	S(c)	S(m)	S(c/m1)	S(c/m2)	S(c/m3)
Ethanol	200	-	-	286	496	549	-	-	292	519	>600
	250	-	-	252	277	180	-	-	281	355	349
	300	126	19	197	106	87	14	90	178	182	142
	350	115	41	127	289	430	26	271	246	235	226
	400	54	56	152	210	295	37	201	467	467	462
Benzene	200	-	-	>600	>600	>600	-	-	>600	>600	>600
	250	-	-	360	486	>600	-	-	60	158	>600
	300	-	-	110	225	403	-	-	14	12	13
	350	-	-	411	422	478	-	-	89	63	78
	400	-	-	173	218	293	-	-	207	103	115
Hydrogen	200	-	-	344	343	349	-	-	84	216	233
	250	-	-	261	162	146	-	-	387	406	404
	300	83	131	96	82	87	194	>600	423	451	442
	350	80	144	87	110	121	165	>600	568	>600	>600
	400	89	34	74	105	110	139	>600	507	519	520
